# Glucocorticoid-Induced TNFR-Related Protein Stimulation Reverses Cardiac Allograft Acceptance Induced by CD40-CD40L Blockade

**DOI:** 10.1155/2013/986859

**Published:** 2013-04-17

**Authors:** Kenneth T. Krill, Keri Csencsits-Smith, Sherri C. Wood, Susan Faust, Guanyi Lu, D. Keith Bishop

**Affiliations:** ^1^Program in Cellular and Molecular Biology, University of Michigan, Medical School, Ann Arbor, MI 48109, USA; ^2^Department of Pathology and Laboratory Medicine, University of Texas Medical School at Houston, Houston, TX 77030, USA; ^3^Section of Transplantation Surgery, Department of Surgery, University of Michigan, Medical School, Ann Arbor, MI 48109, USA; ^4^Graduate Program in Immunology, University of Michigan, Medical School, Ann Arbor, MI 48109, USA

## Abstract

CD40-CD40L blockade has potent immunosuppressive effects in cardiac allograft rejection but is less effective in the presence of inflammatory signals. To better understand the factors that mediate CD40-CD40L blockade-resistant rejection, we studied the effects of stimulation through glucocorticoid-induced TNFR-related protein (GITR), a costimulatory protein expressed by regulatory and effector T cells. Stimulation of CD40−/− or wild-type recipient mice treated with anti-CD40L mAb (WT+anti-CD40L) and with agonistic anti-GITR mAb resulted in cardiac allograft rejection. GITR stimulation did not induce rejection once long-term graft acceptance was established. In vitro, GITR stimulation increased proliferation of effector T cells and decreased regulatory T cell (T_reg_) differentiation in both treatment groups. GITR-stimulated CD40−/− recipients rejected their allografts more rapidly compared to GITR-stimulated WT+anti-CD40L recipients, and this rejection, characterized by a robust Th2 response and significant eosinophilic infiltrate, could be mediated by CD4+ T cells alone. In contrast, both CD4+ and CD8+ T cells were required to induce rejection in GITR-stimulated WT+anti-CD40L-treated recipients, and the pathology of rejection was less severe. Hence, early GITR stimulation could initiate graft rejection despite CD40 deficiency or anti-CD40L mAb treatment, though the recipient response was dependent on the mechanism of CD40-CD40L disruption.

## 1. Introduction

 CD40-CD40L blockade has potent immunosuppressive effects in graft rejection, and an anti-CD40L mAb (MR1) has been shown to induce long-term graft acceptance in mouse cardiac allograft models [1, 2]. Similarly, host CD40 deficiency (CD40−/−) also allows for acceptance of cardiac allografts [3]. Although the mechanisms of allograft acceptance induced by CD40-CD40L blockade are not fully defined, evidence suggests a role for the generation of allograft-specific regulatory T cells (T_reg_) [4, 5]. However, CD40-CD40L blockade is less effective under certain conditions, possibly due to the actions of other costimulatory molecules or the presence of memory T cells [5, 6]. For example, C57BL/6 mice deficient in both CD28 and CD40L acutely reject skin grafts [7, 8], but this rejection can be prevented by blocking OX40-OX40L interactions [7]. Conversely, inductive OX40 stimulation under the cover of CD40-CD40L blockade induces acute cardiac graft rejection, which correlates with the induction of Th1 and Th2 responses as well as the deposition of IgG1 and IgG2a within the graft [9]. Of note, once graft acceptance is established following CD40-CD40L blockade, delayed OX40 stimulation does not induce acute allograft rejection despite priming of graft-reactive Th1 and Th2 cells. However, signs of chronic rejection are observed [9]. Hence, T cell costimulatory pathways other than CD40-CD40L play a role in transplant rejection, though the extent of their influence may be dependent on the inflammatory state of the transplanted tissue (reviewed in [5]).

The glucocorticoid-induced TNFR-related protein (GITR) is a transmembrane receptor belonging to the TNF receptor superfamily and is expressed constitutively at low levels on naive T cells (reviewed in [10]). Following TCR activation, GITR is upregulated on CD4+ and CD8+ T cells. In CD4+ T cells GITR expression may be dependent on CD28 engagement [11, 12], whereas the interplay between CD28 and GITR costimulatory pathways in CD8+ cells has not been fully defined. GITR is also expressed at high levels on T_reg_ and was formerly assumed to be a specific marker for this subset [13]. Studies of agonistic anti-GITR mAb (DTA-1) stimulation showed strong proinflammatory effects in mouse models of autoimmunity, tumor immunity, and infection [11, 14]. The effects of GITR signaling appear to be multifactorial; stimulation through GITR has been demonstrated to increase activation and proliferation of effector T cells (T_eff_), render T_eff_ less resistant to regulation, stimulate inflammatory cytokine secretion by innate immune cells, and increase leukocyte extravasation [11]. Interestingly, GITR stimulation also results in loss of T_reg_ suppressor function, though this effect is transient and appears to be offset in part by the capacity of GITR-stimulated T_reg_ to proliferate [13, 15]. In contrast, blocking GITR interactions through GITR-Fc treatment has been shown to reduce inflammation [16–18]. Therefore, activation through GITR may play a pivotal role in lymphocyte response to transplantation under early inflammatory conditions by affecting the balance between T_eff_ and T_reg_ responses [5]. 

We investigated the consequences of increased GITR activation on graft acceptance in mouse cardiac allograft models based on recipient CD40 deficiency (CD40−/−) or treatment of wild-type recipients with anti-CD40L mAb (WT+anti-CD40L). In vitro, evidence suggested that stimulation through GITR mediated graft rejection both by increasing proliferation of T_eff_ and by inhibiting development of T_reg_. Stimulation through GITR reversed allograft acceptance in both of these models. Interestingly, CD40−/− recipients demonstrated a more severe graft rejection response that could be mediated by CD4+ cells alone, while both CD4+ and CD8+ cells were required to mediate rejection in GITR-stimulated WT+anti-CD40L recipients. Stimulation through GITR was unable to mediate transplant rejection once long-term acceptance of the graft was established. Together, these results demonstrate the capacity of peritransplant GITR stimulation to override the protective effects of CD40-CD40L blockade and highlight the differences in cellular responses caused by CD40 deficiency versus anti-CD40L mAb treatment.

## 2. Materials and Methods

### 2.1. Culture Medium

RPMI supplemented with 2% fetal calf serum, 1 mM sodium pyruvate, 100 U/mL penicillin, 100 *μ*g/mL streptomycin, 1.6 mM L-glutamine, 10 mM HEPES buffer (all from Invitroge, Carlsbad, CA, USA), 0.27 mM L-asparagine, 1.4 mM L-arginine HCl, 14 *μ*M folic acid, and 50 *μ*M 2-ME (all from Sigma-Aldrich, St. Louis, MO, USA).

### 2.2. Mice

WT C57BL/6 (H-2^b^) and BALB/c (H-2^d^) mice of age 6–12 weeks were obtained from Charles River Laboratories (Wilmington, MA, USA). CD40−/− C57BL/6 mice were procured from Jackson Laboratories (Bar Harbor, ME, USA). Breeder pairs of CD40−/− BALB/c mice were kindly provided by Dr. Randolph Noelle (Dartmouth Medical School, Hanover, NH, USA). Foxp3 green fluorescent protein (GFP) reporter knock-in mice bearing a Foxp3-GFP fusion construct were obtained from Dr. Xian Li (Harvard Medical School, Boston, MA, USA; [19]). All animals were maintained under a protocol approved by the University of Michigan Committee on Use and Care of Animals.

### 2.3. Heterotopic Cardiac Transplantation

WT C57BL/6 and CD40−/− C57BL/6 mice were transplanted with WT BALB/c or CD40−/− BALB/c cardiac allografts, respectively. Transplantation of cardiac allografts was performed as previously described [20]. Transplant function was monitored by daily palpation of the graft, and rejection was defined as the cessation of palpable contractions.

### 2.4. Antibodies

Anti-GITR mAb (DTA-1) was generously provided by Dr. Anita Chong (University of Chicago, IL, USA) with kind permission from Dr. Shimon Sakaguchi (Kyoto University, Japan). Purified rat IgG (Sigma-Aldrich) was utilized as an isotype control for anti-GITR. The anti-CD40L mAb producing hybridoma MR1 was obtained from Dr. Randolph Noelle (Dartmouth Medical School). Hybridomas producing anti-CD4 mAb (GK1.5) and anti-CD8 mAb (2.43) were obtained from ATCC (Manassas, VA, USA). All antibodies were produced, purified, and suspended in PBS by LigoCyte Pharmaceuticals, Inc. (Bozeman, MT, USA). Animals receiving anti-CD40L were injected intraperitoneally (i.p.) with 1 mg of mAb on days 0, 1, and 2 relative to transplant. Animals receiving anti-GITR were injected i.p. with 1 mg mAb on days −2 and −1 relative to transplant. For delayed GITR stimulation, anti-GITR mAb was injected at days 29 and 30 after transplant. Anti-CD4 or anti-CD8 mAb were given i.p. on days −1, 0 and 7 relative to transplant at 1 mg/injection. 

### 2.5. Enzyme-Linked Immunospot (ELISPOT) Assay

ELISPOT assays were performed as previously described [21]. Capture and detection of mAb specific for mouse IFN*γ* (R4-6A2, XMG1.2) and IL-4 (11B11, BVD6-24G2) were obtained from BD Biosciences (San Diego, CA, USA). Irradiated (1000 rad) WT or CD40−/− BALB/c splenocytes were added at 4 × 10^5^ cells/well, followed by 1 × 10^6^ recipient WT or CD40−/− splenocytes. After overnight incubation, plates were washed, and biotinylated detection mAb was added, followed by a 1/1000 dilution of polyclonal alkaline phosphatase-conjugated anti-biotin antibodies (Vector Laboratories, Burlingame, CA, USA) in the IFN*γ* wells and a 1/2000 dilution of horseradish peroxidase (HRP) conjugated streptavidin (Dako, Carpinteria, CA, USA) in the IL-4 wells. Plates were developed with NBT/BCIP (IFN*γ*) or 3-amino-9-ethylcarbazole (IL-4). Developed plates were digitally scanned and analyzed using an ImmunoSpot ELISPOT analyzer (Cellular Technologies, Cleveland, OH, USA). 

### 2.6. [^3^H] Thymidine Proliferation Assay

Freshly isolated naïve C57BL/6 responder splenocytes were cultured with irradiated stimulator BALB/c splenocytes with or without 100 *μ*g/mL anti-CD40L mAb (MR1), and naïve C57BL/6 CD40−/− responder splenocytes were cultured with irradiated stimulator BALB/c CD40−/− splenocytes for 5 days. Control rat IgG and anti-GITR mAb were added as indicated at a concentration of 100 *μ*g/mL. 16 hours prior to harvest, cells were pulsed with 0.25 *μ*Ci of [^3^H] thymidine. [^3^H] thymidine incorporation was determined via a Wallac BetaPlate scintillation counter (PerkinElmer, Waltham, MA, USA). Stimulation index was defined as the counts per minute (cpm) of responder cells + stimulator cells divided by the cpm of responder cells only.

### 2.7. In Vitro Generation of T_reg_

Splenocytes from naïve Foxp3 GFP knock-in mice were isolated and cultured for 3 days with 10 U/mL recombinant IL-2, 10 ng/mL recombinant TGF-*β*, and 2% final volume of hybridoma supernatant containing anti-CD3 mAb (YCD3-1). In a modification of a previously published protocol, anti-GITR mAb or rat IgG control Ab was added at a concentration of 100 *μ*g/mL [22]. Lymphocytes were isolated using Ficoll-Paque PLUS (Stemcell Technologies, Vancouver, BC, Canada), stained with phycoerythrin-conjugated anti-CD25 mAb (PC61), and analyzed on a FACSCalibur flow cytometer (BD Biosciences, San Jose, CA, USA).

### 2.8.  Statistical Analyses

Graft survival times were compared using a logrank comparison test. ELISPOT analyses were performed using a Student's *t*-test with Welch's correction (to account for different variances in treatment groups). Proliferation responses were compared using a paired Student's *t*-test. All analyses were performed using GraphPad Prism (GraphPad Software, Inc., La Jolla, CA, USA).

## 3. Results

### 3.1. Anti-GITR mAb Reverses Graft Acceptance Induced by CD40-CD40L Blockade

**Figure 1:**
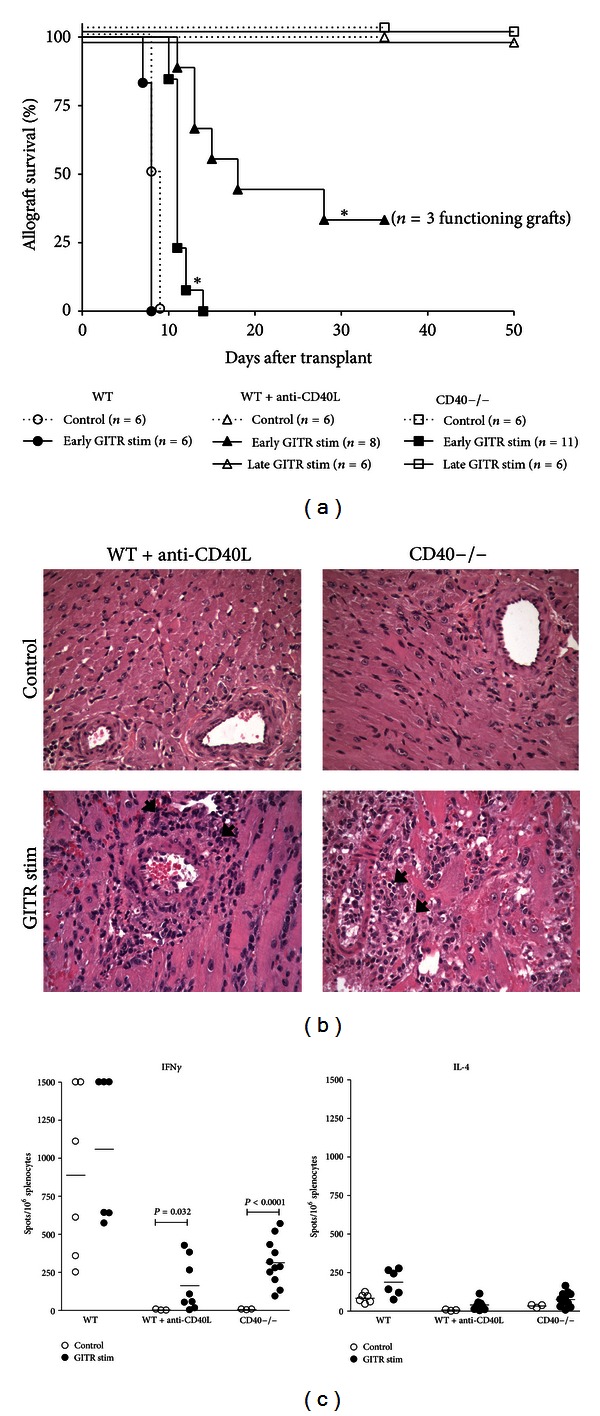
GITR stimulation induces graft rejection in WT+anti-CD40L and CD40−/− allograft recipients. (a) Mann-Whitney survival plot of grafts transplanted into WT (circles), WT+anti-CD40L (triangles), or CD40−/− recipients (squares). All recipients were treated with 1 mg of IgG isotype control (open symbols, dotted line), anti-GITR mAb (closed symbols, solid line) on days −2 and −1 relative to transplantation, or anti-GITR mAb that was administered at days 29 and 30 after transplant (open symbols, solid lines), and mice were observed until 50 days after transplant. Significance was determined via logrank analysis *P < 0.001. (b) Hematoxylin and eosin (H&E; ×200) staining of cardiac allografts recovered from recipients at the day of rejection or at 35 days after transplant (controls). Recipients were treated with either 1 mg of rat IgG isotype (control) or anti-GITR mAb (GITR stim) on days −2 and −1 relative to transplant. Arrows indicate mononuclear cellular infiltrate. (c) Recipient splenocytes were harvested and processed at the time of rejection or at 35 days after transplant for ELISPOT assays, and primed, donor-reactive IFN-*γ* and IL-4 producing cells were quantified as the number of spots/10^6^ splenocytes. Significance was determined by a Student's *t*-test with Welch's correction.

We hypothesized that stimulation through GITR by DTA-1 mAb would exacerbate acute graft rejection and override the protective effects of CD40-CD40L blockade. First, we determined the effects of GITR stimulation on an unmodified WT allogeneic rejection response. C57BL/6 recipients of BALB/c cardiac allografts rejected their grafts by day 7 after transplant, and GITR stimulation did not alter the rate of rejection (Figure 1(a)) or appreciably change allograft histopathology (data not shown). We then tested whether GITR stimulation could reverse allograft acceptance induced by CD40-CD40L blockade. As expected, recipients treated with control rat IgG and anti-CD40L mAb (WT+anti-CD40L) and control rat IgG-treated CD40−/− recipient mice accepted their allografts until the termination of the experiment at 35 days after transplant, demonstrating the effectiveness of CD40-CD40L blockade in inducing graft acceptance. Histology of these allografts was unremarkable and was characterized by a lack of graft infiltrating cells, absence of arterial inflammation, and an abundance of viable nucleated myocytes (Figure 1(b)). 

In contrast, GITR stimulation induced graft rejection in both WT+anti-CD40L and CD40−/− recipients. Interestingly, the rate of rejection was not consistent between the two recipient groups. One hundred percent of the CD40−/− recipients receiving anti-GITR rejected their allografts by day 15 after transplant (Figure 1(a)). Graft pathology in these recipients was characterized by the presence of interstitial hemorrhage, myocyte necrosis, and significant periarteriolar cellular infiltrate (Figure 1(b)). Anti-GITR mAb stimulated rejection in WT+anti-CD40L recipients was less severe, as 66% of mice rejected their grafts before day 35 after transplant (Figure 1(a); median survival 18 days). Analysis of graft pathology revealed moderate periarteriolar mononuclear infiltrate but no significant myocyte necrosis (Figure 1(b)).

Variations in Th cell responses specific to GITR stimulated WT+CD40L or CD40−/− recipients might be responsible for the different rates of rejection. To test this, we utilized ELISPOT assays to quantify graft reactive Th1 (IFN*γ*) and Th2 (IL-4) cells from recipient mice (Figure 1(c)). In both GITR-stimulated WT+anti-CD40L and CD40−/− recipients, graft rejection was characterized by a small but significant increase in IFN*γ* versus recipients treated with control rat IgG. It should be noted that the magnitude of the Th1 response in both GITR-stimulated WT+anti-CD40L and CD40−/− recipients was markedly reduced compared to the Th1 response in WT unmodified recipients, reflecting the ability of CD40-CD40L perturbation to reduce the Th1 response even in the presence of GITR stimulation. The magnitude of Th2 response as measured by IL-4 secretion was unremarkable in both treatment groups. Together, these results suggest that stimulation through GITR is capable of overriding CD40-CD40L perturbation and inducing graft rejection, most likely via activation of donor reactive Th1.

### 3.2. GITR Stimulation Does Not Induce Rejection of Established Allografts

We further investigated the ability of GITR stimulation to induce transplant rejection in established allografts. We have previously demonstrated that OX40 simulation overrides the protective effects of CD40-CD40L blockade when given at the time of transplant. However, delayed OX40 stimulation does not induce acute rejection, instead, signs of chronic rejection are observed [9]. Therefore, we investigated the effects of delayed GITR stimulation on established allografts in WT+anti-CD40L and CD40−/− recipients. Mice received cardiac transplants and were stimulated with 1 mg of anti-GITR mAb on days 29 and 30 after transplant. As demonstrated in Figure 1(a), stimulation of GITR in recipients with established grafts did not result in graft rejection. No inflammatory infiltrate, vascular changes, or obvious differences in collagen deposition were observed between grafts isolated from WT+anti-CD40L and CD40−/− recipients that received late GITR stimulation (data not shown). Investigation of T cell cytokine responses also revealed unremarkable levels of both IFN*γ* and IL-4 in WT+anti-CD40L and CD40−/− recipients that received delayed GITR stimulation (data not shown). Together, these results indicate that stimulation through GITR at the time of transplantation, but not after graft acceptance, overrode the protective effects of CD40L blockade or CD40 deficiency. This indicates that inflammation induced as a consequence of the transplant procedure may play an important role in reversal of graft acceptance by GITR stimulation under the cover of CD40-CD40L blockade. 

### 3.3. GITR Stimulation Modifies Alloantigen-Specific T Cell Proliferation and T_reg_ Differentiation

**Figure 2:**
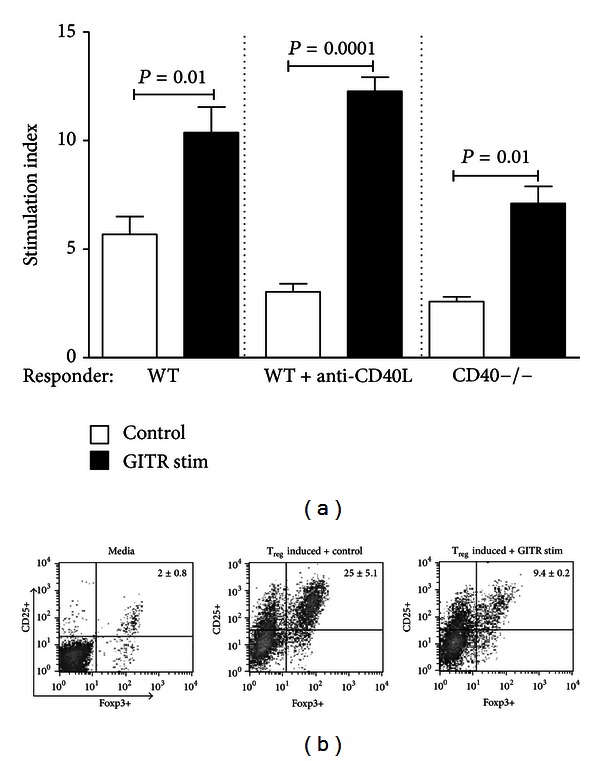
GITR stimulation increases graft-reactive T_eff_ cell proliferation and inhibits T_reg_ development. (a) WT C57BL/6 splenocytes were cultured in the presence of irradiated WT BALB/c splenocytes (WT) or with irradiated WT BALB/c splenocytes + 100 *μ*g/mL anti-CD40L mAb (WT+anti-CD40L); CD40−/− C57BL/6 splenocytes were cultured with irradiated CD40−/− BALB/c splenocytes. Cultures were maintained in the presence of 100 *μ*g/mL of anti-GITR mAb (black bars) or control IgG (open bars) for 5 days and were assayed for proliferation by [^3^H] thymidine incorporation. Stimulation index was defined as responder + stimulator cpm/responder alone cpm. Average values generated from 3 independent experiments are depicted, and significance was determined via paired Student's *t*-test. (b) Naïve splenocytes were isolated from Foxp3-GFP knock-in mice and were cultured for 72 hours in media (left panel), in media containing 10 U/mL recombinant IL-2, 10 ng/mL recombinant TGF-*β*, and 2% anti-CD3 mAb (T_reg_ inducing media) + 100 *μ*g/mL IgG isotype control (middle panel), or in T_reg_ inducing media + 100 *μ*g/mL anti-GITR mAb (right panel). Examples of CD25+ versus Foxp3 GFP+ cell staining from 3 independent experiments are pictured along with the average % staining ± SEM.

Since GITR is expressed on both T_eff_ and T_reg_ [11], we next investigated whether the effects of GITR stimulation in our CD40-CD40L blockade models were due to proliferation of T_eff_ and/or inhibition of T_reg_ development. We characterized the proliferative effect of GITR stimulation on alloreactive splenocytes by utilizing mixed lymphocyte [^3^H] thymidine incorporation assays. Freshly isolated WT responder cells were cocultured with irradiated BALB/c splenocytes only, with anti-CD40L, and irradiated BALB/c splenocytes, and CD40−/− responder cells were cocultured with irradiated CD40−/− BALB/c splenocytes. As depicted in Figure 2(a), stimulation via GITR significantly increased proliferation in all treatment groups relative to isotype-treated control. Clearly, GITR stimulation resulted in an increased T_eff_ response, even under the cover of CD40-CD40L blockade.

We next assessed the ability of GITR stimulation to inhibit T_reg_ differentiation in vitro by utilizing lymphocytes isolated from transgenic mice expressing the Foxp3-GFP fusion protein [19]. Freshly isolated Foxp3-GFP splenocytes were stimulated with anti-CD3 mAb and were cocultured in the presence of TGF*β* and IL-2 to promote differentiation of naïve splenocytes into T_reg_ [23, 24]. Up to 30% of naïve splenocytes were induced to differentiate into CD25+Foxp3+ T_reg_ under these culture conditions (Figure 2(b)). Stimulation through GITR, however, significantly reduced the percentage of CD25+Foxp3+ cells, indicating inhibition of T_reg_ differentiation in vitro. Together, these results suggest that rejection triggered by stimulation through GITR may result from a combination of T_eff_ proliferation together with reduced differentiation of T_reg_.

### 3.4. CD4+ T Cells Are Sufficient to Mediate Rejection in Anti-GITR-Treated CD40−/− Recipients

**Figure 3:**
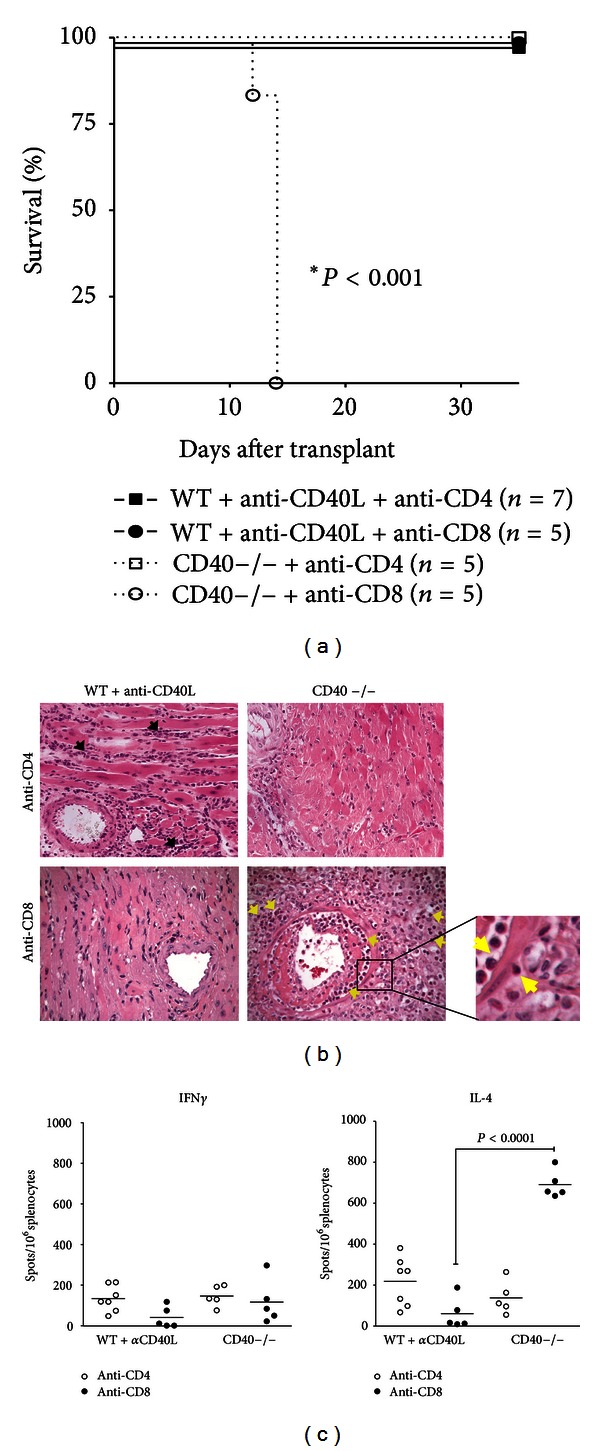
CD4+ T cells mediate GITR-stimulated graft rejection in CD40−/− recipients. (a) Mann-Whitney survival plot of grafts was transplanted into WT+anti-CD40L (closed symbols) or CD40−/− (open symbols) recipients depleted of either CD4+ (squares) or CD8+ (circles) T cells and treated with anti-GITR mAb on days −1 and −2 prior to transplant. Significance was determined via logrank analysis. (b) H&E staining (×200) of transplants recovered either at the day of rejection or at the termination of the experiment at day 35 after transplantation. Black arrows indicate mononuclear cellular infiltrate, and yellow arrows indicate eosinophils. The inset represents 1000x magnification of infiltrate observed in CD8-depleted CD40−/− recipients. (c) Recipient splenocytes were harvested and processed at the time of rejection or at 35 days after transplant for ELISPOT assays, and primed, donor-reactive IFN-*γ* and IL-4 producing cells were quantified as the number of spots/10^6^ total splenocytes. Significance was determined by a Student's *t*-test with Welch's correction.

CD4+ and CD8+ T cells have been shown to be differentially sensitive to various immunomodulatory agents in allograft rejection [25]. Therefore, we determined the requirements for CD4+ and CD8+ T cell subsets in GITR-mediated rejection induced in CD40−/− or WT+anti-CD40L recipients. Depletion of CD4+ T cells in both WT+anti-CD40L and CD40−/− recipients prevented graft rejection induced by GITR stimulation (Figure 3(a)). Depletion of CD4+ T cells resulted in interstitial mononuclear cell infiltrate in grafts; however, cardiac monocytes remained viable (Figure 3(b)). CD8+ T cell depletion in WT+anti-CD40L recipients also prevented GITR-stimulated allograft rejection (Figure 3(a)). Functional grafts recovered from these recipients showed negligible signs of inflammation around myocytes and arterioles (Figure 3(b)). In contrast, allograft rejection occurred within 15 days after transplant following depletion of CD8+ T cells in CD40−/− GITR-stimulated recipients (Figure 3(a)). Grafts isolated from these mice exhibited significant eosinophilic infiltrate extending from the periarteriolar regions of the graft into the surrounding myocytes (Figure 3(b), inset). Extensive loss of viable myocytes, interstitial hemorrhage, and arteriolar occlusion were also evident. The presence of eosinophils within the graft infiltrate suggested induction of a Th2 response [26, 27]. Confirmation of this was achieved via ELISPOT analyses, where significant IL-4, but minimal IFN*γ* production, was observed in GITR-stimulated CD8-depleted CD40−/− splenocytes (Figure 3(c)). Hence, CD4+ T cells alone were capable of rejecting allografts in GITR-stimulated CD40−/− recipients, but both CD4+ and CD8+ T cells are required for GITR stimulation to override the protective effects of anti-CD40L mAb treatment.

## 4. Discussion

This study demonstrates that stimulation through GITR can initiate graft rejection under the cover of CD40-CD40L blockade, though the magnitude of the response depends on the mode of CD40-CD40L blockade and the T cell subset present. Allograft rejection triggered by GITR stimulation in mice with whole T cell populations was characterized by inflammatory cell infiltration and Th1 cytokine secretion (Figure 1). In addition, stimulation through GITR expressed on naïve T cells resulted in enhanced proliferation of WT, WT+anti-CD40L, and CD40−/− responder cells in mixed lymphocyte reactions (Figure 2(a)), demonstrating the ability of signaling through GITR to expand alloantigen-reactive T_eff_ populations. These results thereby reaffirm the costimulatory function of GITR-GITR ligand interactions in the development of an immune response [13, 28]. Thus, despite the protective effects of CD40-CD40L blockade, GITR stimulation and expansion of graft-reactive T_eff_ may simply overwhelm T_reg_-mediated suppression. At the same time, stimulation through GITR may negatively modulate T_reg_ function, further preventing the development of graft acceptance [29, 30]. Indeed, we also noted that stimulation through GITR significantly reduced the development of Foxp3+CD25+ T_reg_ in vitro (Figure 2(b)). 

Importantly, we observed significant differences in the magnitude of rejection responses induced by GITR stimulation in CD40−/− recipients versus WT+anti-CD40L recipients. CD40 deficiency combined with GITR stimulation resulted in a more robust rejection response. Previous evidence has demonstrated that anti-CD40L treatment may have effects beyond simple CD40-CD40L blockade. One possibility is the capacity of anti-CD40L mAb to bind to CD40L expressed on activated T cells, leading to their removal via complement or Fc*γ*R1-mediated mechanisms [31, 32]. Indeed, the effector cells generated in our experiments exhibit a polarized Th1 phenotype (Figure 1(c)), and CD40L expression is enhanced and prolonged on Th1 cells [33]. Therefore, the stimulation of T cells via GITR results in the upregulation of CD40L, and these activated cells might be targeted for clearance by anti-CD40L mAb binding. 

More evidence of increased susceptibility of CD40−/− recipients to GITR stimulation was provided by our finding that CD4+ T cells alone could reject allografts in this setting. In the absence of modifying Th1 cytokines produced by CD8+ T cells, stimulation of CD4+ T cells through GITR resulted in a Th2 response characterized by significant IL-4 production and eosinophilic infiltrate of the graft (Figures 3(b) and 3(c)). This pathogenic Th2 response has previously been associated with CD8+ T cell depletion, IFN*γ* deficiency, or IL-12 antagonism [26, 27, 34, 35]. Thus GITR stimulation can compensate for the lack of CD40 signaling through CD40L on CD4+ T cells. In contrast, anti-CD40L mAb binding to CD40L expressed by activated CD4+ T cells in WT recipients likely results in clearance of these cells via Fc-mediated mechanisms [31, 32]. Hence, the population of cells most responsive to GITR stimulation is absent, and rejection by CD4+ T cells alone cannot occur under the cover of anti-CD40L mAb treatment. 

Importantly, responsiveness to anti-GITR treatment in both WT+anti-CD40L and CD40−/− recipients does not appear to persist for a long term, as GITR stimulation 30 days after transplant did not induce rejection in either recipient strain (Figure 1(a)). This finding is intriguing especially in regards to the WT+anti-CD40L recipients, as these recipients have been shown to retain quiescent donor-reactive T cells in their spleens [1, 36], and these cells could presumably be stimulated by anti-GITR treatment. GITR stimulation likely acts in concert with the inflammatory environment present during early graft-reactive T cell activation. GITR may be upregulated in response to CD28 perturbation in CD4+ T cells [11]; therefore, stimulation through GITR at the time of transplantation likely targets effector T cells that have become activated in response to the graft. In addition, it has been demonstrated that stimulation through GITR results in loss of T_reg_ function (Figure 2(b) and [13]). Therefore, early stimulation through GITR may override the protective effects of CD40-CD40L blockade by providing additional costimulatory signals to T cells and tipping the balance of T_eff_ versus T_reg_. However, once acceptance has been established and allograft inflammation has subsided, GITR stimulation is not sufficient to activate T cells and induce graft rejection. Interestingly, OX40 stimulation under the cover of CD40-CD40L blockade also failed to induce graft rejection once acceptance was established [9], providing further evidence that multiple, inflammatory signaling pathways early in the anti-transplant response may contribute to rejection.

In conclusion, this study has demonstrated the ability of peritransplant GITR stimulation to reverse cardiac allograft acceptance under the cover of CD40-CD40L blockade. These findings reinforce the potential costimulatory role of GITR within the inflammatory environment found after transplant and suggest that stimulation through GITR might inhibit the differentiation of T_reg_ while simultaneously expanding the T_eff_ population. We also demonstrated a marked difference between CD40 deficiency and anti-CD40L mAb treatment in the rate and pathology of graft rejection. These results support reports of the potential pleiotropic effects of anti-CD40L mAb in modulating immune responses [31, 32, 36–39] and suggest that antagonism of GITR signaling might represent a potential therapy for acute inflammatory responses in transplant. 

## Abbreviations

GITR: Glucocorticoid-induced TRFR-related protein
T_reg_: Regulatory T cell
T_eff_: Effector T cell
GFP: Green fluorescent protein
i.p.: Intraperitoneally
ELISPOT: Enzyme-linked immunospot.
